# MPL W515L/K Mutations in Chronic Myeloproliferative Neoplasms

**DOI:** 10.4274/tjh.65807

**Published:** 2013-03-05

**Authors:** Timur Selçuk Akpınar, Veysel Sabri Hançer, Meliha Nalçacı, Reyhan Diz-Küçükkaya

**Affiliations:** 1 İstanbul University, İstanbul Faculty of Medicine, Department of Internal Medicine, Division of Hematology, İstanbul, Turkey; 2 İstanbul Bilim University, Faculty of Medicine, Department of Medical Biology and Genetics, İstanbul, Turkey; 3 İstanbul Bilim University, Faculty of Medicine, Department of Internal Medicine, Division of Hematology, İstanbul, Turkey

**Keywords:** MPL W515L/K mutations, JAK-2 V617F mutation, Myeloproliferative neoplasms, Essential thrombocythemia, Primary myelofibrosis

## Abstract

**Objective:** The MPL gene encodes the thrombopoietin receptor. Recently MPL mutations (MPL W515L or MPL W515K) were described in patients with essential thrombocythemia (ET) and primary (idiopathic) myelofibrosis (PMF). The prevalence and the clinical importance of these mutations are not clear. In the present study, we aimed to investigate the frequency and clinical significance of MPL W515L/K mutations in our patients with ET and PMF.

**Materials and Methods:** A total of 77 patients (66 were diagnosed with ET and 11 with PMF) and 42 healthy controls were included in the study. Using peripheral blood samples, the presence of MPL W515L/K mutations and JAK-2 V617F mutation were analyzed by real-time polymerase chain reaction.

**Results:** In our study, MPL W515L/K or JAK-2 V617F mutations were not observed in healthy controls. JAK-2 V617F mutation was present in 35 patients, of whom 29 had ET (43.9%, 29/66) and 6 had PMF (54.5%, 6/11). In the patient group, MPL W515L/K mutations were found in only 2 PMF cases, and these cases were negative for JAK-2 V617F mutation. The prevalence of MPL W515L/K mutations in the patient group was 2.6%, and the prevalence of MPL W515L/K mutations among the cases negative for the JAK-2 V617F mutation was found to be 4.8%. The 2 cases with MPL W515L/K mutations had long follow-up times (124 months and 71 months, respectively), had no thrombotic or hemorrhagic complications, and had no additional cytogenetic anomalies.

**Conclusion:** MPL W515L/K mutations may be helpful for identifying clonal disease in MPN patients with no established Ph chromosome or JAK-2 V617F mutation.

**Conflict of interest:**None declared.

## Abstract (tr)

**Amaç:** MPL geni trombopoietin reseptörünü kodlamaktadır. Son yıllarda ET ve PMF hastalarında MPL mutasyonları tanımlanmıştır (W515L veya W515K). Bu mutasyonların sıklığı ve klinik önemi henüz net şekilde anlaşılmış değildir. Çalışmamızda, ET ve PMF hastalarında bu mutasyonların sıklığını ve klinik önemini araştırmayı amaçladık.

**Gereç ve Yöntemler:** Çalışmaya toplam 77 hasta (66 ET ve 11 PMF) ve 42 sağlıklı kontrol bireyi dahil edildi. MPL W515L/K ve JAK-2 V617F mutasyon varlığı periferik kan örnekleri kullanılarak gerçek zamanlı polimeraz zincir reaksiyonu yöntemi ile analiz edildi.

B**ulgular:** Sağlıklı kontrol bireylerinde W515L/K veya JAK-2 V617F mutasyonu saptanmadı. 29’u ET (%43,9, 29/66) ve 6’sı PMF (%54,5, 6/11) olan 35 hastada JAK-2 V617F pozitif saptandı. MPL W515L/K mutasyonları JAK-2 V617F negatif olan yalnızca 2 PMF olgusunda pozitif saptandı. MPL W515L/K sıklığı toplam hasta grubunda %2,6, JAK-2 V617F negatif hastalarda ise %4,8 olarak hesaplandı. MPL W515L/K pozitif olan bu 2 hastada herhangi bir trombotik veya kanama komplikasyonu gelişmemiş, sitogenetik anomali saptanmamış, 124 ve 71 ay olan uzun sağkalımları hesaplanmıştır.

**Sonuç:** W515L/K mutasyon varlığı, JAK-2 V617F negatif olan veya Ph kromozomuna sahip olmayan MPN hastalarında klonalitenin saptanmasında yardımcı olabilir.

## INTRODUCTION

Myeloproliferative neoplasms (MPNs) consist of various clonal hematological diseases that are considered to develop from the hematopoietic stem cell transformation. Overproduction of mature, functional blood cells and a chronic clinical course are the main characteristics of these diseases. Chronic myeloid leukemia (CML) is a MPN that is characterized by the development of the Philadelphia (Ph) chromosome and the BCR-ABL fusion gene. Ph-negative MPNs are polycythemia vera (PV), essential thrombocythemia (ET), and primary (idiopathic) myelofibrosis (PMF). The basic feature of these 3 MPNs is that they show hypercellularity in the bone marrow (an increase in the erythrocyte mass in PV, an increase in the platelet number in ET, and advanced fibrosis in the bone marrow in PMF). Individuals with these conditions have a tendency toward both thrombotic and hemorrhagic complications, and are at risk for leukemic transformation [1,2]. 

Janus kinase-2 (JAK-2) is a cytoplasmic tyrosine kinase and has an important role in intracellular signal transduction in hematopoietic cells through the erythropoietin, thrombopoietin (TPO), interleukin-3, granulocyte colony stimulating factor, and granulocyte-macrophage colony stimulating factor receptors. In 2005, an acquired point mutation in the JAK-2 gene (JAK-2 V617F mutation) was discovered in BCR-ABL-negative MPN patients [3]. The JAK-2 V617F mutation occurs as a result of a valine-to-phenylalanine substitution at codon 617 and increases the activity of the growth factor receptor. Through sensitive methods, the presence of the JAK-2 V617F mutation was observed in 90%-95% of patients with PV, in 50%-60% of patients with ET, and in 40%-50% of patients with PMF [3,4]. In JAK-2 V617F mutation-negative-PV patients, other mutations such as JAK-2 exon-12 mutations have been found with relatively low frequency (3%) [5].

Sequence analysis of the MPL gene coding TPO receptor led to discovery of a new molecular abnormality in JAK-2 mutation-negative MPN patients. MPL mutations are located in the juxtamembrane region of the receptor. The most common MPL mutations are W515L (tryptophan-to-leucine substitution) and W515K (tryptophan-to-lysine substitution) [6,7,8]. These mutations can be found in 0% to 10% of PMF patients and in 0% to 6% of ET cases in different series [9,10,11,12]. MPL mutations cause both cytokine-independent growth and hyper-TPO sensitivity in cell lines, primarily by means of activating JAK-STAT/ERK/Akt signal pathways. Other MPL mutations (MPL W515S, W5151A, and MPL S505N) have also been reported in cases of hereditary thrombocytosis [13]. 

In the present study, we aimed to investigate the frequency and clinical significance of MPL W515L/K mutations in our patients with ET and PMF. A total of 77 patients (66 with ET and 11 with PMF) and 42 healthy individuals were analyzed for MPL W515L/K mutations. 

## MATERIALS AND METHODS

**Patients and Controls**

This study was approved by the Ethics Committee of İstanbul University, İstanbul Faculty of Medicine (No: 2009-1976) and was carried out in accordance with the principles of the Helsinki Declaration. The patient group was chosen from among cases followed by the Hematology Division. A total of 77 patients (66 with ET and 11 with PMF) who were followed between 1980 and 2010 and were diagnosed with MPN according to the World Health Organization (WHO) criteria [1], and were negative for the Ph chromosome and/or the BCR-ABL fusion gene, were included in the study. As a control group, 42 healthy individuals who worked at the hospital were analyzed. Personal background details, thrombosis history, physical examination findings, blood counts, peripheral smears, serum iron status (ferritin levels, iron, and total iron-binding capacity values), bone marrow aspiration, biopsy details, and cytogenetic analyses were screened and recorded electronically. 

**Methods**

Peripheral venous blood was taken from all subjects. The presence of the MPL W515 mutations was screened using melting curve analysis at the molecular hematology laboratory. All samples were analyzed for JAK-2 V617F mutation and MPL W515L/K mutations. Genomic DNA was isolated from peripheral venous blood leukocytes using a spin-colon DNA isolation kit (Roche Diagnostics, Mannheim, Germany). Genotyping was performed using melting curve analysis with LightCycler 2.0 (Roche Diagnostics). Specific primers and probes were designed using Primer Express 3.0 software. 

For the JAK-2 V617F LightCycler assay, polymerase chain reaction (PCR) was carried out in capillaries in a total reaction volume of 20 µL containing 25 ng of genomic DNA, 200 µmol/L of dNTPs, 4 mmol/L of MgCl2, 0.1 µmol/L of forward primer (TTCCTTAGTCTTTCTTTGAAGGT), 0.5 µmol/L of reverse primer (GTGATCCTGAAACTGAATTTTCT), and 0.2 µmol/L each of the sensor (5^'^-ATGGAGTATGTGTCTATGGAGTATGTGTCTGTGG-fluorescein-3^'^) and anchor (5^'^-LCR640-ACGAGAGTAAGTA AAACTACAGGTC- phosphate-3^'^) probes using the following PCR program: initial denaturation at 95 ^°^C for 10 min and 45 amplification cycles at 55^°^C for 10 s and 72 ^°^C for 10 s. Melting analysis was performed by denaturing at 95 ^°^C for 30 s and cooling to 35 ^°^C for 1 min, followed by heating at the rate of 0.1 °C/s from 35 °C to 80 ^°^C. Melting peaks generated with the LightCycler displayed a distinct melting temperature for the wild type (60 ^°^C) and the mutant (52 ^°^C). 

For the W515 mutations, PCR reactions included 25 ng genomic DNA, 200 µmol/L dNTP, 4 mmol/L MgCl2, 0.2 µmol/L forward primer (TGGGCCGAAGTCTGACCCTTTA), 0.5 µmol/L reverse primer (ACAGAGCGAACCAAGAATGCCTGTG), and 0.2 µmol/L anchor probe (LC640-AGGCCCAGGACGGCGT) and sensor probe (CTGCCACCTCAGCAGCAT-fluorescein-3'). The PCR procedure was performed with 45 amplification cycles of 10 min of denaturation at 95 ^°^C, 10 s at 55 ^°^C, and 10 s at 72 ^°^C. Melting curve analysis was performed with 30 s of denaturation at 95 ^°^C and 1 min of cooling at 35 °C, followed by heating to 85 °C at a speed of 0.1 ^°^C/s. The results were evaluated by considering the melting temperature. Because the probe was designed to match the non-mutated portion (wild-type) of the target DNA, it had a higher melting temperature (62.5 ^°^C). MPL W515 mutation-positive samples melted at a lower temperature (52.5 ^°^C). 

In evaluating the findings obtained in the study, NCSS (Number Cruncher Statistical System) 2007 and PASS 2008 Statistical Software (Utah, USA) were used for the statistical analyses. 

## RESULTS

In our study, MPL W515L/K and JAK-2 V617F mutations were not found in healthy controls. In the patient group, the JAK-2 V617F mutation was present in a total of 35 patients, 29 of which had ET and 6 of which had PMF. The frequency of JAK-2 V617F mutation in ET patients was 43.9%, and in PMF patients it was 54.5%. 

MPL W515L/K mutation was found in 2 cases. Both cases were from the PMF group and were negative for JAK-2 V617F mutation. The prevalence of the MPL W515 L/K mutations was found to be 2.6% (2/77) in the patient group, 4.8% (2/42) in patients who were negative for JAK-2 V617F mutation, and 18.2% in the PMF group (2/11). The clinical, laboratory, and molecular characteristics of the PMF patients found to have a MPL W515 mutation are presented in Table 1.

**Figure 1:**
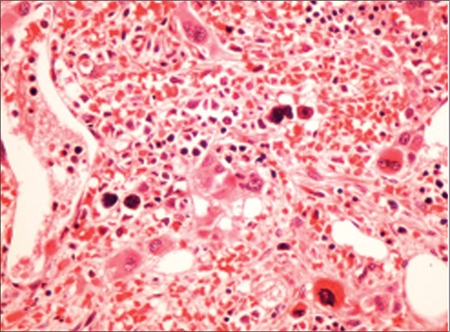
Dysplastic megakaryocytes with mononuclear and hypolobulated forms (hematoxylin and eosin, 400^×^).

Patient 1, a 62-year-old male patient, complained of abdominal swelling and weakness. Upon physical examination, it was determined that he had hepatomegaly extending beyond the rib curve by 6 cm and splenomegaly extending beyond the rib curve by 10 cm. The patient had known diagnoses of chronic hepatitis B infection and atrial fibrillation with no history of thrombosis. His peripheral smear revealed 3% blast cells, 2% myelocytes, 4% metamyelocytes, tear drop-shaped erythrocytes, and giant platelets (Figure 1). A bone marrow biopsy revealed hypocellular bone marrow containing dense reticulin fibers (grade 3). Hydroxyurea, warfarin, and allopurinol were used for treatment. Follow-up time was 124 months with no thrombotic or hemorrhagic complications.

**Figure 2:**
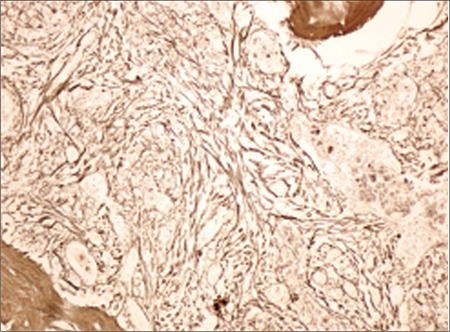
Grade 4 fibrosis shown with reticulin stain (400^×^).

Patient 2, a 58-year-old female patient, presented with complaint of fatigue. Physical examination revealed splenomegaly extending beyond the rib curve by 9 cm. She had no additional disease or history of thrombosis. The peripheral smear revealed erythroblastosis and tear drop-shaped erythrocytes. Hypocellular bone marrow displayed elevated levels of dense reticulin fibers, as shown in Figure 2 (grade 4). Low-dose aspirin, hydroxyurea, and folbiol were used for treatment. Follow-up time was 115 months with no complications.

## DISCUSSION

The presence of MPL mutations in patients with MPN was first published by Pikman et al. in 2006 [6]. Studies including larger cohorts of patients showed different frequencies of MPL mutations in MPN patients. Pardanani et al. [7] screened MPL mutations in 1182 patients with MPN (290 with PMF, 242 with PV, 318 with ET) and other myeloid disorders (88 with myelodysplastic syndrome, 118 with CML, 126 with acute myeloid leukemia), regardless of JAK-2 V617F mutational status. They found that MPL mutations were not present in PV, myelodysplastic syndrome, CML, or acute myeloid leukemia patients; only ET and PMF patients were found to carry these mutations. The frequency of MPL mutations was found to be low in patients with PMF (5%) and ET (1%). They also reported that 6 patients had both JAK-2 V617F and MPL W515L/K mutations [7]. Beer et al. analyzed a PT-1 cohort for MPL mutations and screened for MPL mutations in 776 patients with ET [8]. MPL mutations were found in 32 patients (4.1%). One of these patients also had JAK-2 V617F mutation. They found that patients with MPL mutations had lower hemoglobin levels and higher platelet counts at diagnosis compared with patients with JAK-2 V617F mutation. They did not find any relation between MPL W515L/K mutations and thrombosis, major hemorrhage, fibrotic transformation, or disease duration [8]. Schnittger et al. [12] evaluated 869 MPN patients who were negative for JAK-2 V617F mutation. They found that 35 had MPL W515L/K mutations (26 with ET, 7 with PMF, 1 with chronic myelomonocytic leukemia, and 1 with secondary acute myeloid leukemia after PMF). In their cohort, the frequency of MPL W515L/K mutations was 5.3% in ET and 9.6% in PMF patients [12].

In Asian studies, however, the frequencies of MPL W515L/K mutations were not consistent. Lieu et al. [9] evaluated 88 Taiwanese patients with MPN and could not find any MPL mutations. Ruan et al. [10] analyzed 343 MPN patients negative for JAK-2 V617F mutation. They found that 3.5% of ET (7/199) and 12.5% of PMF (3/24) patients had MPL W515L/K mutations. 

In our study, MPL W515L/K mutations were investigated in 77 patients who were diagnosed with ET and PMF according to the WHO criteria. Forty-two patients were negative for JAK-2 V617F mutation. We found that MPL W515L/K mutations were present in only 2 patients with PMF (18.2%), and both of those patients were negative for JAK-2 V617F mutation. These patients had no thrombotic or hemorrhagic complications and had a long disease course (124 and 115 months). In conclusion, MPL W515L/K mutations may be helpful for identifying clonal disease in MPN patients with no established Ph chromosome or JAK-2 V617F mutation. Further studies including larger cohorts of patients will help to understand the phenotypic effects of these mutations. 

**Conflict of Interest Statement**

The authors of this paper have no conflicts of interest, including specific financial interests, relationships, and/ or affiliations relevant to the subject matter or materials included.

## Figures and Tables

**Table 1:**
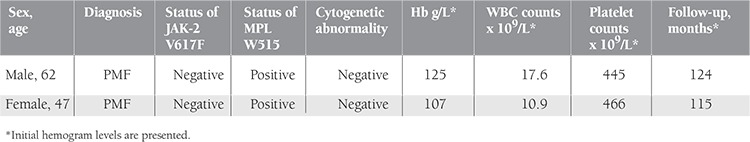
Clinical, laboratory, and molecular characteristics of two patients with MPL W515L/K mutations.
